# Characterization of white matter over 1–2 years in small vessel disease using MR-based quantitative susceptibility mapping and free-water mapping

**DOI:** 10.3389/fnagi.2022.998051

**Published:** 2022-09-30

**Authors:** Yawen Sun, Ying Hu, Yage Qiu, Yuyao Zhang, Changhao Jiang, Peiwen Lu, Qun Xu, Yuting Shi, Hongjiang Wei, Yan Zhou

**Affiliations:** ^1^Department of Radiology, School of Medicine, Ren Ji Hospital, Shanghai Jiao Tong University, Shanghai, China; ^2^School of Information and Science and Technology, ShanghaiTech University, Shanghai, China; ^3^Department of Neurology, School of Medicine, Ren Ji Hospital, Shanghai Jiao Tong University, Shanghai, China; ^4^Ren Ji-UNSW CHeBA Neurocognitive Center, School of Medicine, Ren Ji Hospital, Shanghai Jiao Tong University, Shanghai, China; ^5^Department of Health Manage Center, School of Medicine, Ren Ji Hospital, Shanghai Jiao Tong University, Shanghai, China; ^6^School of Biomedical Engineering, Shanghai Jiao Tong University, Shanghai, China

**Keywords:** small vessel disease, quantitative susceptibility mapping, free-water mapping, white matter lesions, normal-appearing white matter

## Abstract

**Purpose:**

The aim of this study was to investigate alterations in white matter lesions (WMLs) and normal-appearing white matter (NAWM) with small vessel disease (SVD) over 1–2 years using quantitative susceptibility mapping (QSM) and free-water (FW) mapping.

**Methods:**

Fifty-one SVD patients underwent MRI brain scans and neuropsychological testing both at baseline and follow-up. The main approach for treating these patients is the management of risk factors. Quantitative susceptibility (QS), fractional anisotropy (FA), mean diffusivity (MD), FW, FW-corrected FA (FA_T_), and FW-corrected MD (MD_T_) maps within WMLs and NAWM were generated. Furthermore, the JHU-ICBM-DTI label atlas was used as an anatomic guide, and the measurements of the segmented NAWMs were calculated. The average regional values were extracted, and a paired *t*-test was used to analyze the longitudinal change. Partial correlations were used to assess the relationship between the MRI indices changes (e.g., ΔQS_followup − baseline_/QS_baseline_) and the cognitive function changes (e.g., ΔMoCA_followup − baseline_/MoCA_baseline_).

**Results:**

After SVD risk factor control, no gradual cognitive decline occurred during 1–2 years. However, we still found that the QS values (index of demyelination) increased in the NAWM at follow-up, especially in the NAWM part of the left superior frontal blade (SF), left occipital blade, right uncinate fasciculus, and right corticospinal tract (CST). FW (index of neuroinflammation/edema) analysis revealed that the follow-up group differed from the baseline group in the NAWM part of the right CST and inferior frontal blade (IF). Decreased FA_T_ (index of axonal loss) was observed in the NAWM part of the right SF and IF at follow-up. In addition, the FA_T_ changes in the NAWM part of the right IF were associated with overall cognitive performance changes. In contrast, no significant differences were found in the WMLs.

**Conclusion:**

The NAWM was still in the progressive injury process over time, while WMLs remained relatively stable, which supports the notion that SVD is a chronic progressive disease. The process of axonal loss in the NAWM part of the prefrontal lobe might be a biomarker of cognitive changes in the evolution of SVD.

## Introduction

Small vessel disease (SVD) is a common accompaniment of aging. It refers to a syndrome of clinical and MRI findings that are thought to result from pathologies in perforating cerebral arterioles, capillaries, and likely venules (Shi and Wardlaw, 2016). SVD can be accelerated and potentiated by different vascular risk factors (Caruso et al., 2019; Moretti and Caruso, 2020). Cognitive impairment problems are frequently seen in SVD. SVD progresses to subcortical vascular dementia (SVaD) and contributes to 45% of dementia cases (Gorelick et al., 2011). The diagnosis of SVD has heavily relied on non-invasive MRI findings since it is challenging to visualize SVD pathologies *in vivo*. White matter lesions (WMLs) on MRI are considered the hallmark of SVD and have become a significant contributor to cognitive dysfunction (Wardlaw et al., 2013; Shi and Wardlaw, 2016). However, SVD brain damage is not confined to visible lesions. It is a dynamic and whole-brain disease (Shi and Wardlaw, 2016). More sensitive MRI methods indicate that microstructural alterations occur in the surrounding normal-appearing white matter (NAWM), which worsens as WMLs increase (Maniega et al., 2015; Munoz Maniega et al., 2017). Thus, finding potential microstructural changes in NAWM for early diagnosis before the onset of clinical deterioration is an urgent matter.

The pathophysiological mechanisms underlying the development from NAWM to WMLs in SVD remain poorly understood. The underlying pathology of WMLs primarily reflects demyelination, axonal loss, edema, and inflammation (Craggs et al., 2014; Prins and Scheltens, 2015; Rosenberg, 2017). However, pathological studies mainly and inevitably describe late-stage disease, which provides limited insights into the dynamic evolution of tissue changes. Cerebrospinal fluid biomarkers can also provide a more precise measurement of SVD (Paolini Paoletti et al., 2021; Moretti and Caruso, 2022). For example, myelin lipid sulfatide and myelin basic protein may reflect demyelination and remyelination processes. The neurofilament light chain, a marker of axonal damage, was found to increase early in SVD (Paolini Paoletti et al., 2021). However, noninvasive tests would have to be widely performed. To this end, advanced MRI techniques have been used in the *in vivo* assessment of brain tissue and have been shown to reflect the histopathological and functional substrates in WMLs and the NAWM (Promjunyakul et al., 2018; Wong et al., 2019; Konieczny et al., 2021). Previous diffusion tensor imaging (DTI) studies have shown that the development of focal WMLs is preceded by diffuse alterations to the microstructure of the surrounding NAWM (Maillard et al., 2011; de Groot et al., 2013). Diffusion-related changes in the tissue microstructure in NAWM are reflective of progressive damage before the appearance of WMLs, which have significant impact on the rate of cognitive decline (Vemuri et al., 2021). Longitudinal studies have shown that structural integrity changes in the NAWM at baseline are associated WML expansion over time, with decreased fractional anisotropy (FA) and increased mean diffusivity (MD) (Promjunyakul et al., 2018; van Leijsen et al., 2018). At the histopathologic level, the DTI measures (FA and MD) were associated with axonal and myelin density of water matter in SVD (van Veluw et al., 2019). However, vasogenic edema associated with neuroinflammation cannot be measured or differentiated from axon/myelin injury by DTI, limiting its clinical applications. In addition, diffusion measures within brain tissues can be affected by partial volume effects due to edema and cerebrospinal fluid (Alexander et al., 2007; Metzler-Baddeley et al., 2012). When voxels contain free water, diffusion measures can be biased toward a pattern of high MD and reduced FA.

Free-water (FW) mapping is a novel DTI analysis technique obtained from a bi-tensor model. It was developed to explicitly estimate the fractional volume of freely diffusing water molecules within the voxel (Pasternak et al., 2009; Metzler-Baddeley et al., 2012). It can provide more microstructural information because it can separate the diffusion properties of water in brain tissue from those of FW. It enables differentiation between alterations in the tissues themselves as measured by FW-corrected DTI indices and extracellular FW changes, such as neuroinflammation, as measured by the fractional volume of FW (Pasternak et al., 2009; Andica et al., 2019). This technique can provide novel evidence to distinguish between axonal injury and neuroinflammation in the white matter (Wang et al., 2011), which may provide a better understanding of the progression of SVD. Furthermore, quantitative susceptibility mapping (QSM) can be used to quantify myelin levels and thereby demyelination in tissue at the voxel scale, as the magnetic properties of myelin are vastly different from those of axons. The myelin sheath is the predominant source of susceptibility contrast in the white matter, mainly because of its diamagnetic property (Cao et al., 2014; Wang et al., 2019). Thus, QSM could be more sensitive and specific to the demyelination process than diffusion metrics (Liu et al., 2011; Cao et al., 2014). Therefore, QSM together with FW mapping has great potential to provide noninvasive biomarkers for demyelination, neuroinflammation, and axonal injury coexisting in SVD.

In the present longitudinal study, we performed a multiparametric evaluation using QSM and FW mapping in SVD focusing on white matter at baseline and follow-up (two time points with a 1–2-year period). The main approach for treating these SVD patients is the management of risk factors and healthy lifestyle behaviors. Currently, prevention is particularly important when there is no effective treatment for cognitive decline in SVD. Common risk factors, such as diabetes mellitus, hypertension, hyperlipidemia, and cigarette smoking, contribute to SVD changes through the build-up of atherosclerotic plaque, lipohyalinosis, arteriolosclerosis, and fibrinoid necrosis (Raman et al., 2016). We hypothesized that QSM and FW mapping could provide complementary information about the white matter of SVD patients. We compared quantitative susceptibility (QS) values and FW values and examined the effect of FW correction on FA and MD changes in WMLs and NAWMs. The comparisons were performed on two levels: a global white matter analysis and an atlas-based region of interest (ROI) analysis. We performed ROI analysis to investigate the alterations in specific white matter tracts, which may contribute to explaining the relationship with cognitive function. Finally, we explored the association between these MRI indices and cognitive function.

## Materials and methods

### Subjects

All SVD patients were recruited from among patients who were admitted to the Neurology Department of Ren Ji Hospital. Each patient underwent a standard baseline evaluation, including neurological examination, complete sociodemographic and clinical data, including vascular risk factors, neuropsychological tests, and multimodal brain MRI examination. In brief, the recruitment and inclusion criteria were as follows: (1) age between 50 and 85 years old; (2) education over 6 years; (3) at least 1 month after clinical stroke accident; (4) presence of ≥ 1 subcortical lacunar infarct(s) and WMLs on MRI; and (5) a modified Rankin score ≤ 3 points. Exclusion criteria were as follows: (1) severe systemic or other diseases that may cause cognitive dysfunction; (2) cortical and/or cortico–subcortical non-lacunar infarcts; (3) WMLs due to other specific causes; (4) cardioembolic or large-vessel diseases; (5) severe brain atrophy; (6) intracranial space-occupying lesions; (7) microbleeds or hemorrhage; (8) severe depression (17-item Hamilton Depression Rating Scale score ≥ 24); (9) other major central neurological or psychiatric disorders; and (10) severe claustrophobia or contraindications to MRI examination. All the patients were right-handed.

The follow-up neuropsychological tests and brain MRI scans were performed 1–2 years later. Finally, altogether, 51 SVD patients with DTI, QSM, and structural MRI scans and neuropsychological assessments both at baseline and at follow-up were included in this study. No patient suffered a transient ischemic attack or a stroke between baseline and follow-up.

### Neuropsychological tests

A standardized battery of multidomain cognitive tests was performed within a week after MRI examination by a trained neuropsychologist. The MoCA and Mini-Mental State Examination (MMSE) were used to assess overall cognitive performance. In addition, the following tests were used to evaluate four key cognitive domains: (1) attention and executive function: Trail-Making Tests A and B (TMT-A, TMT-B), Stroop color-word test C (Stroop C-T), and category Verbal Fluency Test (VFT); (2) memory function: Rey Auditory Verbal Learning Test of short- and long-delay free recall (AVLT-short, AVLT-long); (3) language function: Boston naming test (30 items) (BNT); and (4) visuospatial function: Rey-Osterrieth Complex Figure Test (copy) (Rey-O copy).

### MRI data acquisition

MRI scanning was conducted at baseline and follow-up both on a 3.0 T MRI scanner (GE Signa HDxt, USA) equipped with an eight-channel phased array head coil at Ren Ji Hospital. No major hardware or software upgrades to the MRI system occurred during the duration of this study. Restraining foam pads were used to reduce head motion, and earplugs were used to reduce scanner noise. QSM images with whole-brain coverage were acquired using a standard flow-compensated three-dimensional spoiled gradient recalled (3D-SPGR) sequence with the following parameters: TE_1_/ΔTE/TE_16_ = 3.2/2.42/39.5 ms, TR = 42.5 ms, FA = 12°, bandwidth = 62.5 kHz, FOV = 220 × 220 mm^2^, matrix = 256 × 256, slices = 66, slice thickness = 2 mm, and spatial resolution = 0.86 × 0.86 × 1 mm^3^. DTI images were acquired using a spin-echo single-shot echo-planar pulse sequence with the following parameters: TE = 89.8 ms, TR = 17,000 ms, FOV = 256 × 256 mm^2^, matrix = 128 × 128, gap = 0, slices = 66, slice thickness = 2 mm, 20 diffusion-weighted scans with a b-value of 1,000 s/mm^2^, and b0 = 0. Sagittal T1-weighted (T1w) images were acquired with a 3D-SPGR sequence with the following parameters: TE = 1.7 ms, TR = 5.5 ms, TI = 450 ms, FA = 15°, FOV = 256 × 256 mm^2^, matrix = 256 × 256, gap = 0, slices = 155, and slice thickness = 1.0 mm. The parameters used to obtain axial T2-fluid attenuated inversion recovery sequence (FLAIR) were as follows: TE = 150 ms, TR = 9,075 ms, TI = 2,250 ms, FOV = 256 × 256 mm^2^, matrix = 256 × 256, slices = 66, and slice thickness = 2 mm.

### Image reconstruction and processing

#### Reconstruction of QSM images

QSM images were reconstructed from GRE phase data using the STISuite toolbox (https://people.eecs.berkeley.edu/~chunlei.liu/software.html). The details of QSM processing have been presented in previous studies (Wei et al., 2015, 2017). Briefly, the 3D phase image from the complex GRE data was first unwrapped using the Laplacian-based method (Schofield and Zhu, 2003). The tissue phase was estimated from the unwrapped image by removing the background phase using variable-kernel sophisticated harmonic artifact reduction for phase data (V_SHARP) (Wu et al., 2012). Finally, a two-step field-to-source method called streaking reduction in QSM (STAR-QSM) (Wei et al., 2015) was used for dipole inversion to calculate the magnetic susceptibility.

#### Diffusion MRI preprocessing

All diffusion MRI data from 64 different axial, sagittal, and coronal directions were visually checked. Moreover, all datasets were free from severe artifacts such as gross geometric distortion, signal dropout, or bulk motion. Diffusion MRI data were then corrected for susceptibility-induced geometric distortions, eddy current distortions, and intervolume subject motion. Single-tensor FA and MD maps were generated using the DTIFIT tool implemented in FMRIB Software Library version 5.0.9 (FSL; Oxford Center for Functional MRI of the Brain, Oxford, UK; www.fmrib.ox.ac.uk/fsl). Moreover, FW maps were calculated by fitting a bi-tensor model based on the diffusion measurements (Ofori et al., 2015a,b; Burciu et al., 2017). The bi-tensor model predicts the signal attenuation of the water molecules contributed by intracellular and extracellular water. FW mostly occupies the extracellular space. The FW maps reflect the volume fraction of FW content within each voxel. The generated FW maps include FW, FA, FW-corrected FA (FA_T_), MD, and FW-corrected MD (MD_T_) maps.

#### Image registration and generation of labels

**Figure 1:**
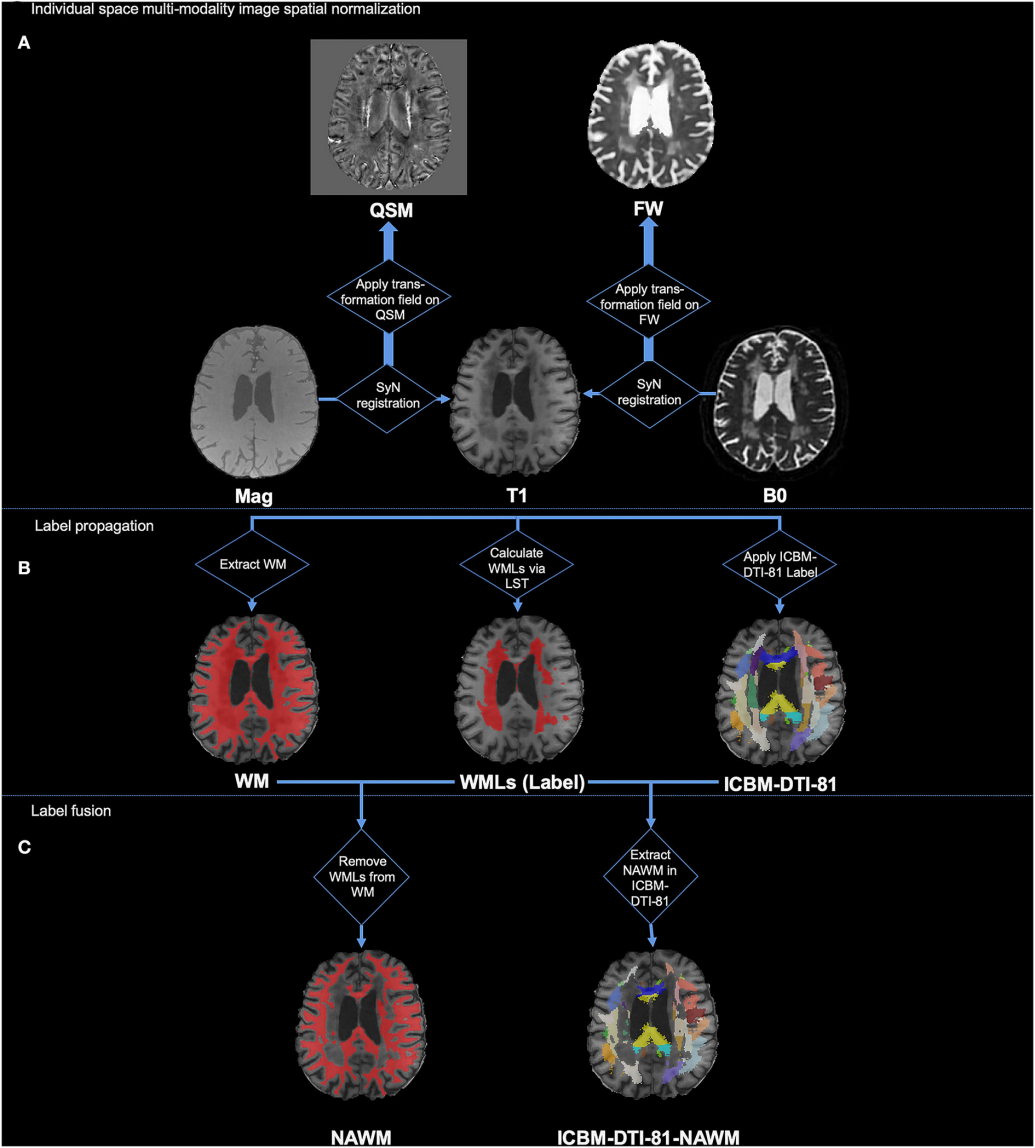
Illustration of processing pipeline of image registration and generation of labels. **(A)** Individual space multi-modality image spatial normalization; **(B)** Label propagation; **(C)** Label fusion.

Figure 1 illustrates the pipeline of registration and generation of labels. First, we used the T1w image as the intermedium to register the multimodal images into the same space. For each individual, the magnitude image was registered to the T1w image using SyN registration. The generated deformation field was then used to register the QSM image to the T1w image. Similarly, the B0 image was registered to the T1w image using SyN registration, and then, the deformation field was applied to register the FW, FA, FA_T_, MD, and MD_T_ images to the T1w image. Second, we registered the individual T1w images to the MNI standard space using SyN registration, and the inverse deformation field was applied to register ICBM-DTI-81 labels from the MNI standard space to the individual T1w space.

After the registration of the multimodal images and sets of labels, we then used them to generate the WMLs label, NAWM label, and ICBM-DTI-81-NAWM labels. Specifically, the ICBM-DTI-81-NAWM labels were the WMLs removed from white matter fiber bundle parcellation, as shown in Figure 1C label fusion part. First, the segment of the white matter mask was generated from the individual T1w image by FAST in FSL (Zhang et al., 2001; Jenkinson et al., 2012). Second, the T1w image and the FLAIR image of each individual were used to delineate the WMLs (WMLs ROI in Figure 1B) in T1w space using the LST's lesion growth algorithm (Schmidt et al., 2012). We gave it the “WMLs” label. Afterward, we removed the WMLs ROI from the white matter mask and provided this fused mask with the “NAWM” label. In addition, we assessed the quality of the automatic labeling of WMLs and NAWM. By intersecting the NAWM mask and the ICBM-DTI-81 labels, we finally generated the ICBM-DTI-81-NAWM labels, which indicated the NAWM part in each of the white matter tracts. Finally, values in each ROI of T1w, QSM, and FW maps (e.g., mean, volume) of the WML label, NAWM label, and ICBM-DTI-81-NAWM labels were calculated for further statistical analysis.

### Statistical analyses

All statistical analyses were performed using SPSS (IBM SPSS Statistics v23.0; IBM Co., Armonk, NY, USA) and MATLAB R2012b (MathWorks, Natick, MA, USA). A paired *t*-test was used to analyze the longitudinal changes, and the evaluated aspects included the mean values and volumes of WMLs, NAWM, and the NAWM part in each of the white matter tracts. Comparisons between the baseline and follow-up clinical scores were also performed with a paired *t*-test. The average regional values were extracted to perform partial correlation analyses to assess whether cognitive function was associated with the white matter regions exhibiting significant differences in MRI indices. Delta values were calculated as the degree of change between the follow-up and baseline (e.g., ΔQS_followup − baseline_/QS_baseline_). Associations between longitudinal MRI indices changes (e.g., ΔQS_followup − baseline_/QS_baseline_) and cognitive function changes (e.g., ΔMoCA_followup − baseline_/MoCA_baseline_) were explored using partial correlation analysis, with patient age, gender, years of education, and mean follow-up time as covariates. *P* < 0.05 was considered statistically significant in this study (without multiple comparison correction as an explorative study).

## Results

### Demographic and clinical characteristics

Table 1 shows baseline demographic characteristics, risk factors, and cognitive test score differences between baseline and follow-up. Fifty-one SVD patients were included in this study, with a mean age of 64.69 ± 6.53 years, and 43 SVD patients were male (84.31%). There were no significant differences in MMSE, MoCA, TMT-A, Stroop C-T, VFT, AVLT-short, AVLT-long, BNT, or Rey-O copy scores between baseline and follow-up (all *p* > 0.05). However, the follow-up group had a lower TMT-B score than the baseline group (baseline: 188.98 ± 76.37 s; follow-up:174.16 ± 70.44 s; *p* = 0.024). For the TMT-B, the lower score reveals executive function improvement. In our study, the TMT-B scores at baseline and follow-up were both within the normal range (the deficient score should be >273 s). The mean follow-up time was 1.19 ± 0.42 years (from 0.84 to 2.40 years).

**Table 1:** Demographic and clinical characteristics of the participants.

**SVD *n* = 51**	**Baseline**	**Follow-up**	***p*-Values**
Demographic factors			
Age (mean ± SD)	64.69 ± 6.53	/	/
Gender ratio (male %)	84.31%	/	/
Education years (mean ± SD)	10.10 ± 2.18	/	/
Risk factors			
Diabetes mellitus (%)	37.25%	/	/
Hypertension (%)	78.43%	/	/
Hyperlipidemia (%)	21.60%	/	/
Cigarette smoking (%)	50.98%	/	/
Neuropsychological tests			
MoCA	23.45 ± 3.80	22.86 ± 4.25	0.244
MMSE	27.75 ± 1.98	27.49 ± 2.61	0.476
TMT-A	81.96 ± 38.14	76.94 ± 49.77	0.346
TMT-B	188.98 ± 76.37	174.16 ± 70.44	**0.024**
Stroop C-T	109.53 ± 50.26	102.43 ± 43.87	0.237
VFT	14.59 ± 3.51	14.33 ± 4.00	0.604
AVLT-short	5.06 ± 2.05	5.41 ± 2.26	0.215
AVLT-long	4.33 ± 2.71	5.35 ± 4.81	0.054
BNT	23.55 ± 3.40	23.80 ± 3.76	0.513
Rey-O copy	32.41 ± 6.09	32.92 ± 5.46	0.631
Mean follow-up time in years, (mean ± SD)	/	1.19 ± 0.42	/

SVD, small vessel disease; SD, standard deviation; MoCA, Montreal Cognitive Assessment; MMSE, Mini-Mental State Examination; TMT-A, Trail-Making Tests A; TMT-B, Trail-Making Tests B; Stroop C-T, Stroop color-word test C; VFT, category Verbal Fluency Test; AVLT-short, Rey Auditory Verbal Learning Test of short-delay free recall; AVLT-long, Rey Auditory Verbal Learning Test of long-delay free recall; BNT, Boston naming test (30 items); Rey-O copy, Rey-Osterrieth Complex Figure Test (copy).

p-value < 0.05 was considered statistically significant (highlighted in bold).

### MRI indices in the WMLs and NAWM regions between baseline and follow-up

**Figure 2:**
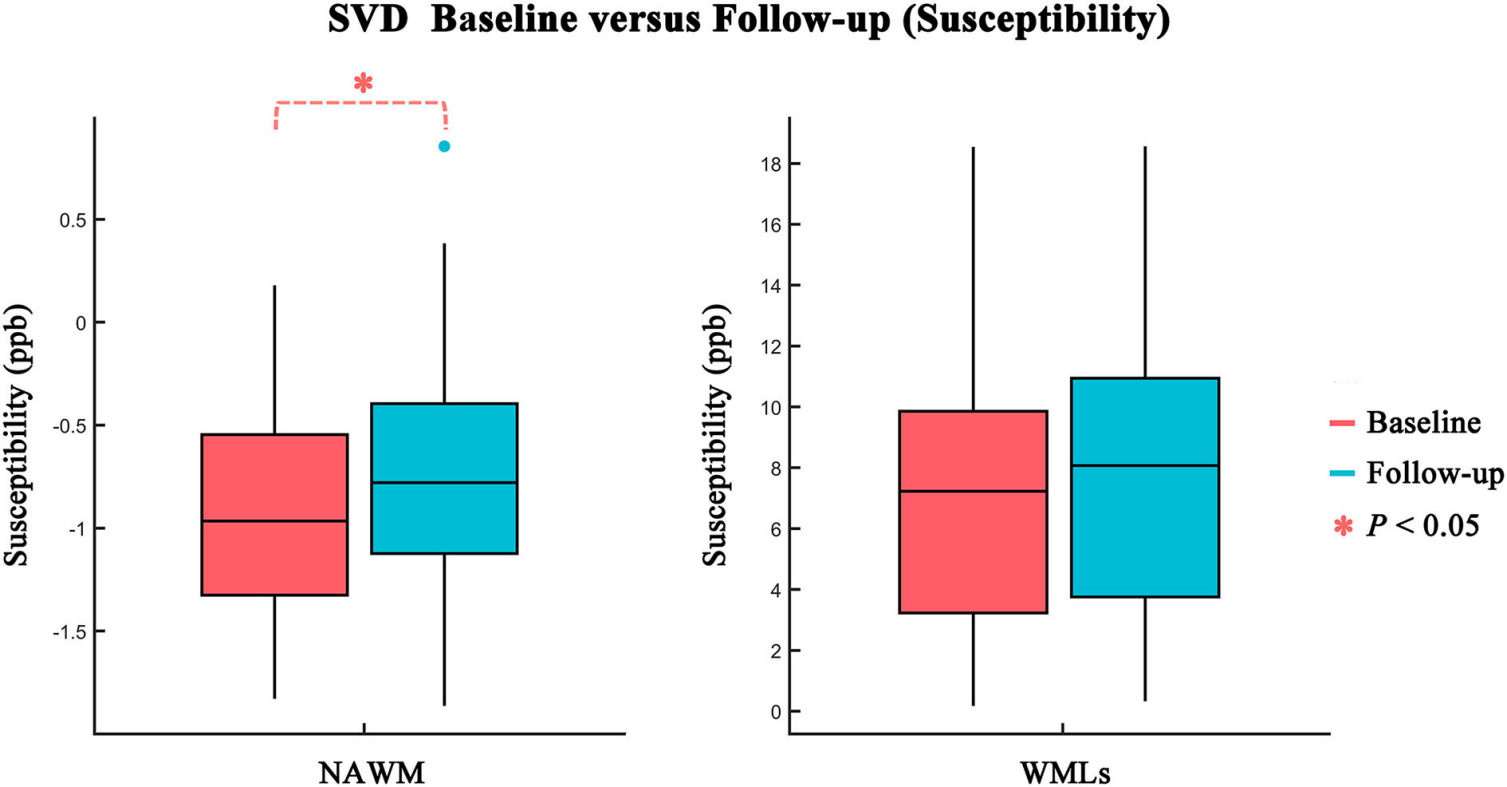
Comparison of susceptibility values between baseline (red) and follow-up (blue) in NAWM and WMLs. Statistically significant difference is delineated by red dashed bracket.

The comparisons between baseline and follow-up in WMLs and NAWM regions are summarized in Table 2 and Figure 2. Over 1–2 years, no significant difference was found in QS, FA, MD, FW, FA_T_, or MD_T_ values or in the volumes between baseline and follow-up in the WMLs region (all *p* > 0.05). However, a significant difference in QS values was found in NAWM. The mean QS values increased significantly at follow-up (baseline: −0.876 ± 0.534 ppb; follow-up: −0.748 ± 0.574 ppb; *p* = 0.029). No differences in the other MRI indices outlined above were found when comparing baseline and follow-up for NAWM.

**Table 2:** Baseline and follow-up MRI indices in WMLs and NAWM in SVD.

	**NAWM**		***p*-Value**	**WMLs**		***p*-Value**
	**Baseline**	**Follow-up**		**Baseline**	**Follow-up**	
QS (ppb)	−0.876 ± 0.534	−0.748 ± 0.574	**0.029**	7.048 ± 4.411	8.017 ± 4.744	0.086
FW	0.341 ± 0.020	0.345 ± 0.019	0.167	0.371 ± 0.027	0.376 ± 0.033	0.270
FA	0.331 ± 0.016	0.329 ± 0.017	0.253	0.367 ± 0.079	0.383 ± 0.072	0.146
FAt	0.407 ± 0.024	0.403 ± 0.025	0.055	0.509 ± 0.111	0.525 ± 0.110	0.313
MD (× 10^−3^mm^2^s^−1^)	0.901 ± 0.052	0.906 ± 0.052	0.357	1.024 ± 0.092	1.026 ± 0.114	0.900
MDt (× 10^−3^mm^2^s^−1^)	0.653 ± 0.059	0.663 ± 0.058	0.109	0.700 ± 0.094	0.707 ± 0.111	0.557
Volume (cc)	598.146 ± 54.194	598.474 ± 59.233	0.918	5.268 ± 7.224	5.193 ± 7.022	0.599

WMLs, white matter lesions; NAWM, normal-appearing white matter; SVD, small vessel disease; QS, quantitative susceptibility; FW, free water; FA, fractional anisotropy; Fat, FW-corrected FA; MD, mean diffusivity; MDt, FW-corrected MD.

p-value < 0.05 was considered statistically significant (highlighted in bold).

### MRI indices in the NAWM part of the white matter tracts between baseline and follow-up

**Figure 3:**
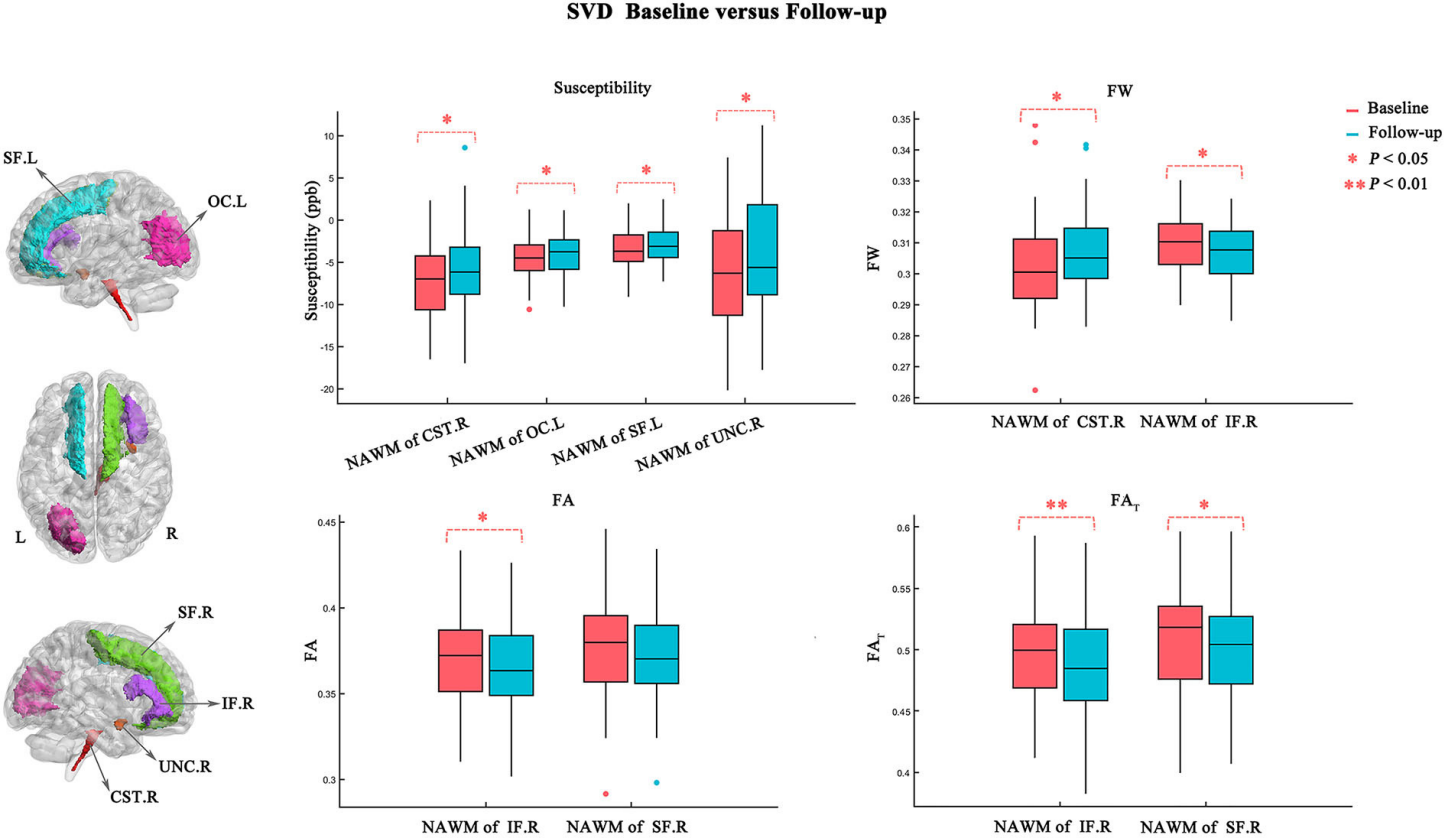
Atlas-based analysis results for susceptibility, FW, FA, and FA_T_ values between baseline (red) and follow-up (blue). Statistically significant difference is delineated by red dashed bracket.

Figure 3 and Table 3 show the atlas-based analysis results for QS values, volume, DTI (FA and MD), and FW imaging indices (FW, FA_T_, and MD_T_) between baseline and follow-up. Among the NAWM parts of the white matter tracts, the left superior frontal blade (baseline: −3.454 ± 2.148 ppb; follow-up: −2.861 ± 2.004 ppb; *p* = 0.041), left occipital blade (baseline: −4.411 ± 2.518 ppb; follow-up: −3.800 ± 2.455 ppb; *p* = 0.022), right uncinate fasciculus (baseline: −6.481 ± 6.720 ppb; follow-up: −4.265 ± 7.267 ppb; *p* = 0.033), and right corticospinal tract (baseline: −7.134 ± 4.388 ppb; follow-up: −5.623 ± 4.549 ppb; *p* = 0.044) demonstrated significantly higher QS values at follow-up than at baseline. The results of FW analysis revealed that SVD patients at follow-up had higher FW in the NAWM part of the right corticospinal tract (baseline: 0.302±0.015; follow-up: 0.307 ± 0.013; *p* = 0.029) and lower FW in the NAWM part of the right inferior frontal blade (baseline: 0.310 ± 0.010; follow-up: 0.307±0.009; *p* = 0.021). The right inferior frontal blade demonstrated significantly decreased FA (baseline: 0.372 ± 0.028; follow-up: 0.367 ± 0.027; *p* = 0.027) and FA_T_ (baseline: 0.498 ± 0.041; follow-up: 0.488 ± 0.042; *p* = 0.009) at follow-up compared to baseline. Moreover, the SVD patients had decreased FA_T_ in the right superior frontal blade (baseline: 0.510 ± 0.042; follow-up: 0.503 ± 0.041; *p* = 0.011) at follow-up. No significant differences in MD, MD_T_, or volume were found in this longitudinal study.

**Table 3:** Baseline and follow-up MRI indices in the NAWM part in each of the WM tracts in SVD.

	**Supeiror.frontal.blade.L**		***p*-Value**	**Occipital.blade.L**		***p*-Value**
	**Baseline**	**Follow-up**		**Baseline**	**Follow-up**	
QS (ppb)	−3.454 ± 2.148	−2.861 ± 2.004	**0.041**	−4.411 ± 2.518	−3.800 ± 2.455	**0.022**
FW	0.313 ± 0.015	0.312 ± 0.013	0.425	0.307 ± 0.018	0.309 ± 0.017	0.32
FA	0.379 ± 0.029	0.378 ± 0.028	0.428	0.353 ± 0.031	0.348 ± 0.032	0.151
FAt	0.515 ± 0.043	0.511 ± 0.041	0.195	0.455 ± 0.043	0.447 ± 0.046	0.093
MD (× 10^−3^mm^2^s^−1^)	0.844 ± 0.052	0.841 ± 0.047	0.442	0.838 ± 0.055	0.843 ± 0.053	0.396
MDt (× 10^−3^mm^2^s^−1^)	0.543 ± 0.055	0.543 ± 0.047	0.96	0.561 ± 0.055	0.568 ± 0.055	0.207
Volume (cc)	8.859 ± 1.008	8.952 ± 1.089	0.124	8.850 ± 1.245	9.035 ± 1.235	0.104
	**Uncinate.fasciculus.R**		***p*** **-Value**	**Corticospinal.tract.R**		***p*** **-Value**
	**Baseline**	**Follow-up**		**Baseline**	**Follow-up**	
QS (ppb)	−6.481 ± 6.720	−4.265 ± 7.267	**0.033**	−7.134 ± 4.388	−5.623 ± 4.549	**0.044**
FW	0.329 ± 0.017	0.330 ± 0.014	0.917	0.302 ± 0.015	0.307 ± 0.013	**0.029**
FA	0.489 ± 0.057	0.488 ± 0.067	0.958	0.497 ± 0.050	0.498 ± 0.042	0.869
FAt	0.669 ± 0.075	0.670 ± 0.091	0.95	0.675 ± 0.063	0.679 ± 0.054	0.443
MD (× 10^−3^mm^2^s^−1^)	0.845 ± 0.072	0.843 ± 0.057	0.869	0.744 ± 0.049	0.755 ± 0.047	0.128
MDt (× 10^−3^mm^2^s^−1^)	0.507 ± 0.059	0.507 ± 0.052	0.969	0.441 ± 0.044	0.450 ± 0.044	0.104
Volume (cc)	0.251 ± 0.036	0.246 ± 0.040	0.218	1.177 ± 0.181	1.147 ± 0.180	0.517
	**Inferior.frontal.blade.R**		***p*** **-Value**	**Superior.frontal.blade.R**		***p*** **-Value**
	**Baseline**	**Follow-up**		**Baseline**	**Follow-up**	
QS (ppb)	−0.045 ± 2.356	0.040 ± 2.577	0.788	−2.973 ± 2.043	−2.781 ± 1.803	0.46
FW	0.310 ± 0.010	0.307 ± 0.009	**0.021**	0.314 ± 0.013	0.314 ± 0.014	0.736
FA	0.372 ± 0.028	0.367 ± 0.027	**0.027**	0.377 ± 0.028	0.374 ± 0.028	0.087
FAt	0.498 ± 0.041	0.488 ± 0.042	**0.009**	0.510 ± 0.042	0.503 ± 0.041	**0.011**
MD (× 10^−3^mm^2^s^−1^)	0.849 ± 0.038	0.843 ± 0.039	0.066	0.850 ± 0.048	0.849 ± 0.048	0.879
MDt (× 10^−3^mm^2^s^−1^)	0.541 ± 0.035	0.540 ± 0.036	0.868	0.547 ± 0.049	0.551 ± 0.050	0.246
Volume (cc)	5.043 ± 0.705	5.018 ± 0.712	0.685	9.721 ± 1.141	9.677 ± 1.161	0.526

NAWM, normal-appearing white matter; WM, white matter; SVD, small vessel disease; QS, quantitative susceptibility; FW, free water; FA, fractional anisotropy; Fat, FW-corrected FA; MD, mean diffusivity; MDt, FW-corrected MD.

p-value < 0.05 was considered statistically significant (highlighted in bold).

### Correlations between longitudinal MRI indices changes and cognitive function changes

**Figure 4:**
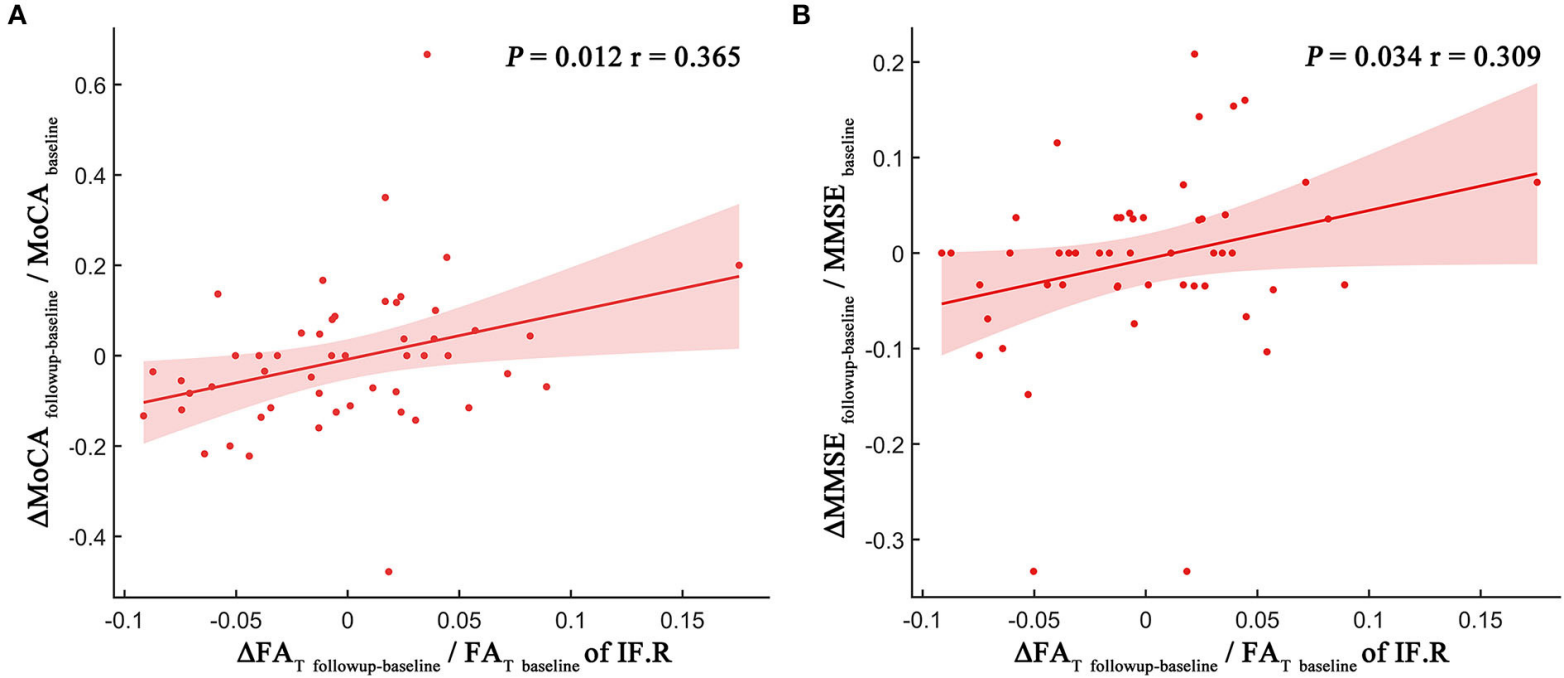
Scatter plot illustrating the relationship between longitudinal MRI indices changes and MoCA score changes **(A)** and MMSE score changes **(B)**. The dots represent the adjusted values after controlling age, gender, years of education, and mean follow-up time.

The degree of the FA_T_ changes (ΔFA_Tfollowup − baseline_/ FA_Tbaseline_) in the NAWM part of the right inferior frontal blade was positively correlated with both MoCA score changes (ΔMoCA_followup − baseline_/MoCA_baseline_) (*p* = 0.012, r = 0.365; Figure 4A) and MMSE score changes (ΔMMSE_followup − baseline_/MMSE_baseline_) (*p* = 0.034, r = 0.309; Figure 4B), which represent overall cognitive performance. However, no significant correlations were identified between other MRI indices and neuropsychological tests.

## Discussion

The current study explored the WMLs and NAWM alterations in patients with SVD using QSM and FW images. Although there is no evidence for cognitive decline over 1–2 years in SVD, the change in QSM and FW indices is detectable in NAWM. First, there was a trend toward increased QS in the NAWM (especially in the NAWM part of the left superior frontal blade, left occipital blade, right uncinate fasciculus, and right corticospinal tract) at follow-up, which usually results from demyelination. Second, the FW values in the follow-up group differed significantly from the baseline group in the NAWM part of the right corticospinal tract and inferior frontal blade, which indicated neuroinflammation and edema changes. Third, decreased FA_T_ was observed in the NAWM part of the right superior frontal blade and inferior frontal blade at follow-up, whereas decreased FA was observed only in the NAWM part of the right inferior frontal blade. The FW-corrected DTI indices may be able to derive more precise estimations of axonal loss compared with the DTI indices. In contrast, there was no significant difference in these MRI indices between baseline and follow-up in the WMLs over 1–2 years. We further found that the FA_T_ changes in the NAWM part of the right inferior frontal blade were associated with overall cognitive performance changes, which indicated that axonal loss in the prefrontal lobe might be a biomarker of cognitive function. Overall, this longitudinal study in SVD has shown that changes in QSM and FW indices are detectable in NAWM over a 1–2-year follow-up period, which illustrated that NAWM was still in the progressive injury process. Our study supported that SVD is a chronic progressive disease.

WMLs are the typical signs of SVD and are usually symmetrically and bilaterally distributed in the white matter. They appear hyperintense to the brain on T2/FLAIR sequences and can be patchy or confluent depending on their stage of development and severity. The neuropathologic substrate of WMLs is a combination of demyelination, inflammation, edema, and loss of axons. Previous studies on WMLs change in SVD were mostly about progression. Both population-based and hospital-based studies have shown that WML volume increases over time (mean follow-up duration: 0.8–11 years) (van Leijsen et al., 2017). More recently, a decrease in WML volume has also been reported (Cho et al., 2015; Ramirez et al., 2016; Al-Janabi et al., 2019), challenging the assumption of a continuous progressive nature of SVD markers. They speculated that the change could be not only to permanent myelin loss or axonal damage but also to shifts in water content. WML regression may represent a longitudinal reduction in neuroinflammatory and edema changes associated with SVD (Wardlaw et al., 2015). In addition, a stable group of WMLs has also been identified in some studies (Ramirez et al., 2016; Al-Janabi et al., 2019). In sum, the WML change in SVD over time should be considered a dynamic and variable process. Our data show that there were no significant differences in WML volume between baseline and follow-up over 1–2 years, as well as no significant differences in FW values (index of neuroinflammation/edema), QS values (index of demyelination), and FW-corrected DTI indices (index of axonal loss). This implies that at least in this longitudinal study, the WMLs in SVD patients remained relatively stable. Thus, we also explored the changes within the NAWM in SVD over the years.

The neuropathologic changes in white matter spread widely, rather than being confined to visible WMLs. The demyelination, axonal loss, edema, and inflammation radiate into NAWM, indicating that the underlying pathology is a diffuse process affecting much of the white matter and that visible WMLs are probably only the “tip of the iceberg” (Wardlaw et al., 2015). Our results demonstrated that the NAWM changes might be more dynamic than previously thought. First, our work supported QSM as a sensitive technique that can specifically measure demyelination in NAWM. SVD is thought to result from chronic and progressive hypoperfusion affected by abnormal cerebral arterioles and capillaries. Tissue hypoxia plays a potential pathogenic role in the development of WMLs. Previous studies have found that hypoperfusion always precedes NAWM changes (Promjunyakul et al., 2016; Wu et al., 2019). Indeed, the cells which are most vulnerable to hypoxic damage in NAWM, including oligodendrocytes (oligodendrocytes form myelin and support axons by supplying energy), die first, resulting in demyelination in SVD (Martinez Sosa and Smith, 2017). QSM has been developed to derive voxel-based magnetic susceptibility. Myelin is the dominant source of susceptibility contrast in white matter. It has been shown that QSM may be more sensitive and specific to the demyelination/remyelination process than DTI metrics (Liu et al., 2011; Wang et al., 2019). In this study, we found increased QS values in the NAWM to be widespread over 1–2 years, which should result from demyelination. We speculated that loss of oligodendrocytes during chronic and progressive hypoperfusion might contribute to demyelination in NAWM and that NAWM in SVD is still in the progressive injury process over the years.

Blood–brain barrier permeability increases with age, and this process appears to be accelerated in SVD (Farrall and Wardlaw, 2009). Several studies have suggested that blood–brain barrier leakage may be positively associated with WMLs (Huisa et al., 2015; Kerkhofs et al., 2021). Blood–brain barrier leakage is apparent in NAWM and worsens with proximity to the WMLs. Blood–brain barrier leakage might play an early role in subsequent microstructural white matter degeneration as part of the pathophysiology of SVD (Kerkhofs et al., 2021). Dysfunction of the blood–brain barrier might have several adverse effects: The leakage of fluids, proteins, and other plasma constituents into the perivascular tissues might increase interstitial fluid (edema) and neuroinflammation (Wardlaw et al., 2019). A recent hypothesis suggested that SVD patients show leakage of toxic plasma components through an impaired blood–brain barrier and cause secondary inflammation and injury to axons and myelin (Wardlaw et al., 2003). In the past, *in vivo* MRI has thus far lacked the specificity to demonstrate neuroinflammation/edema within the NAWM. Currently, FW mapping can address these effects. Our results from FW analysis demonstrated the neuroinflammation/edema alterations in the NAWM part of the corticospinal tract and inferior frontal blade in SVD. This finding indicated that the NAWM might include areas of tissue neuroinflammation/edema, and neuroinflammation/edema might progress or regress over time. The regression in tissue neuroinflammation/edema at a later stage could then lead to reduced WML volume. This might be the reason why WMLs regress in SVD over time. In addition, a recent study using neurite orientation dispersion and density imaging demonstrated that increased FW was the strongest predictor of cognitive function in participants in the Mayo Clinic Study of Aging (Raghavan et al., 2021). However, no significant correlations were identified in our SVD study. Future work should be undertaken to widely validate and compare FW outcomes in a larger patient cohort.

In the present study, we also found decreased FA_T_ in the NAWM part of the right superior frontal blade and inferior frontal blade at follow-up, which is consistent with previous DTI studies (de Laat et al., 2011; Liu et al., 2020). Previous studies demonstrated that the FA_T_ was able to derive more precise estimations of localized white matter damage compared with the DTI indices. The explanation could be that partialling out FW eliminates the influence of water on white matter tracts, thereby increasing the specificity of FW imaging indices (Pasternak et al., 2009; Oestreich et al., 2017; Andica et al., 2019). Moreover, reduced FA_T_ usually results from axonal loss or damage that is permanent. Thus, we speculated that the NAWM of the prefrontal lobe might have suffered permanent and irreversible damage over 1–2 years in SVD. Specifically, our study also found both decreased FW and decreased FA_T_ in the NAWM part of the right inferior frontal blade over time, which might support that neuroinflammation/edema may precede axonal damage in the NAWM/WMLs of patients with SVD. Furthermore, the degree of FA_T_ changes in the prefrontal lobe has been linked to MoCA and MMSE score changes on tests of cognitive function. The impact of white matter damage on cognition is likely to depend not only on their total load but also on their location. Previous studies have pointed out that the prefrontal lobe plays an important role in cognitive control (Miller and Cohen, 2001). In particular, the inferior frontal blade is thought to exert a key role in the executive control of attention (Yin et al., 2013). Several meaningful studies have suggested that the main cognitive impairment is attention/executive function in SVD across multiple domains of cognition (Prins et al., 2005; Liang et al., 2021). Our results showed that white matter damage in the right inferior frontal blade, an important part of the executive/attention system, may lead to the progression of cognitive impairment, consistent with previous studies. Thus, the FA_T_ could derive precise estimations of axonal loss, which was considered permanent and irreversible. We speculated that axonal loss in the NAWM part of the prefrontal lobe might be crucial for the pathophysiology of SVD-related cognitive impairment.

This study has several limitations. First, although SVD is a common brain disease and our group has collected neuroimaging data on SVD for many years, only a few patients were willing to take part in the follow-up study. Thus, only 51 SVD patients with complete MRI scans and neuropsychological assessments both at baseline and at follow-up were included in this study. Given the small sample size, only tentative conclusions can be drawn. In addition, our study lacks a control group. The presence of a control group would be important to draw deeper conclusions. Second, the manifestations of WMLs are complex, and we were unable to determine the precise mechanisms underlying these changes using *in vivo* MRI. Third, our study described the change in SVD markers over time using neuroimaging at only two time points, and the follow-up period was short. More time points will be needed to describe the dynamic changes. Future studies should examine longitudinal changes in a larger patient cohort over a longer time period. Fourth, multiple comparison correction was not applied because we were performing an exploratory analysis. Fifth, our study focused on the white matter of SVD. Future research will explore the differences in gray matter atrophy between baseline and follow-up and associations with other SVD markers.

## Conclusion

In the present study, we determined that NAWM was still in the progressive injury process over 1–2 years, while WMLs remained relatively stable. QSM and FW mapping can be considered specific and sensitive tools for the monitoring of complex pathologies such as demyelination, neuroinflammation/edema, and axon loss in SVD. Furthermore, the process of axonal loss in the NAWM part of the prefrontal lobe, which is considered permanent and irreversible, might be a biomarker for the pathophysiological mechanism of cognitive decline in the evolution of SVD. Our results supported that SVD is a chronic progressive disease. We hope the early identification of pathological characteristics with advanced MR imaging can provide an opportunity to forestall progression before the emergence of symptoms in SVD. More effective therapies are urgently needed in future.

## Data availability statement

The raw data supporting the conclusions of this article will be made available by the authors, without undue reservation.

## Ethics statement

The studies involving human participants were reviewed and approved by the Research Ethics Committee of the Ren Ji Hospital, School of Medicine, Shanghai Jiao Tong University. The patients/participants provided their written informed consent to participate in this study.

## Author contributions

YSu, YQ, and YH performed the MR examination. YSu, CJ, and YSh performed the image analysis. YSu and YH performed the statistical analysis. QX and PL performed neuropsychological tests. YZho and HW participated in the study design. YZho and YZha reviewed the manuscript. YSu was a major contributor in writing the manuscript. All authors read and approved the final manuscript and contributed to the discussion of the manuscript.

## Funding

This work was supported by the National Natural Science Foundation of China (Grant Nos. 81901693, 82171885, and 82001457), Shanghai Rising Stars of Medical Talent Youth Development Program, Youth Medical Talents-Medical Imaging Practitioner Program (Grant No. SHWRS(2020)_087), Shanghai Science and Technology Committee Project (Natural Science Funding: Grant No. 20ZR1433200), Shanghai Science and Technology Committee Project (Explorer Project Funding: Grant No. 21TS1400700), and Medical Engineering Cross Research Foundation of Shanghai Jiao Tong University (Grant No. YG2017QN47).

## Conflict of interest

The authors declare that the research was conducted in the absence of any commercial or financial relationships that could be construed as a potential conflict of interest.

## Publisher's note

All claims expressed in this article are solely those of the authors and do not necessarily represent those of their affiliated organizations, or those of the publisher, the editors and the reviewers. Any product that may be evaluated in this article, or claim that may be made by its manufacturer, is not guaranteed or endorsed by the publisher.
